# Mammary-Enriched Transcription Factors Synergize to Activate the *Wap* Super-Enhancer for Mammary Gland Development

**DOI:** 10.3390/ijms231911680

**Published:** 2022-10-02

**Authors:** Uijin Kim, Suyeon Kim, Nahyun Kim, Ha Youn Shin

**Affiliations:** Department of Biomedical Science and Engineering, Konkuk University, Seoul 05029, Korea

**Keywords:** super-enhancer, ELF5, STAT5, CRISPR-Cas9, mammary gland development

## Abstract

Super-enhancers are large clusters of enhancers critical for cell-type-specific development. In a previous study, 440 mammary-specific super-enhancers, highly enriched for an active enhancer mark H3K27ac; a mediator MED1; and the mammary-enriched transcription factors ELF5, NFIB, STAT5A, and GR, were identified in the genome of the mammary epithelium of lactating mice. However, the triggering mechanism for mammary-specific super-enhancers and the molecular interactions between key transcription factors have not been clearly elucidated. In this study, we investigated in vivo protein–protein interactions between major transcription factors that activate mammary-specific super-enhancers. In mammary epithelial cells, ELF5 strongly interacted with NFIB while weakly interacting with STAT5A, and it showed modest interactions with MED1 and GR, a pattern unlike that in non-mammary cells. We further investigated the role of key transcription factors in the initial activation of the mammary-specific *Wap* super-enhancer, using CRISPR-Cas9 genome editing to introduce single or combined mutations at transcription factor binding sites in the pioneer enhancer of the *Wap* super-enhancer in mice. ELF5 and STAT5A played key roles in igniting *Wap* super-enhancer activity, but an intact transcription factor complex was required for the full function of the super-enhancer. Our study demonstrates that mammary-enriched transcription factors within a protein complex interact with different intensities and synergize to activate the *Wap* super-enhancer. These findings provide an important framework for understanding the regulation of cell-type-specific development.

## 1. Introduction

Mammary gland tissue undergoes a cycle of development and involution processes that occur in sequential stages, starting with the establishment of the ductal tree during puberty and proceeding through the formation of alveoli during pregnancy, lactation, and tissue remodeling upon weaning [1,2]. Mammary gland epithelium, which plays a key role in temporal regulatory dynamics during development, is composed of basal myoepithelial cells and milk-secreting luminal alveolar cells. The lactating alveolar epithelium is formed during pregnancy and becomes remodeled when lactation is stopped. Differentiation of the luminal lineage is primarily regulated by the cytokine prolactin (PRL), which is secreted during pregnancy and lactation [3,4] and leads to the activation of a number of mammary-specific genes [5]. A previous study reported that mammary-specific genes are regulated by 440 super-enhancers that are activated in the lactating mammary gland [6]. A super-enhancer is a cluster of enhancers spanning a long region of genomic loci that is associated with genes involved in cell identity [7,8,9]. Mutations in super-enhancers are also closely associated with a variety of diseases, including cancer, diabetes, and autoimmune diseases [7,10,11,12,13,14]. Integrated chromosome immunoprecipitation sequencing (ChIP-seq) analyses have demonstrated that mammary-specific super-enhancers are co-occupied by histone H3 acetylation at lysine 27 (H3K27ac), a histone mark characteristic of transcriptionally active enhancers, as well as several mammary-enriched transcription factors and mediator complex subunit 1 (MED1) [6]. Mammary-enriched transcription factors include signal transducer and activator of transcription 5A (STAT5A), glucocorticoid receptor (GR), nuclear factor 1 B-type (NFIB), and E74-like factor 5 (ELF5), which play essential roles in mammary alveolar differentiation [15,16,17,18].

Among genes associated with mammary-specific super-enhancers, the expression of *Whey acidic protein* (*Wap)*, a major milk protein gene in mice, is increased over 10,000-fold during lactation [6,15]. The *Wap* super-enhancer is composed of three individual enhancers, E1, E2, and E3 [6]. All three constituent enhancers are co-occupied by the mammary-enriched transcription factors STAT5A, GR, NFIB, and ELF5, as well as MED1, and are distinguished by H3K27ac marks. However, the molecular interactions among these transcription factors are largely unknown. The proximal E1 enhancer plays a pioneer function, serving to activate distal E2 and E3 enhancers [6]. A previous study demonstrated that combined mutations of STAT5, NFIB, and ELF5 binding sites in the E1 enhancer in mice eliminated the capacity to activate the super-enhancer and completely silenced the *Wap* gene. Although a single mutation in the STAT5 binding site or a combined mutation in STAT5 and NFIB binding sites was not sufficient to disrupt *Wap* super-enhancer function, it remained unclear whether binding of ELF5 alone or binding of all three transcription factors is essential for full super-enhancer function.

Here, we investigated protein–protein interactions among ELF5, NFIB, GR, STAT5A, and MED1 within mammary epithelial cells using an in vivo bimolecular fluorescence complementation (BiFC) assay, a method commonly used to establish and quantify in vivo interactions of fluorescent reporter-tagged proteins [19,20,21]. We further verified interactions of these proteins in the lactating mammary epithelium in mice using in situ proximity ligation assays (PLAs). PLAs, which integrate co-immunoprecipitation and co-localization techniques without requiring cell or tissue modification, enable protein interactions of two different proteins to be determined in situ [22,23,24]. Moreover, to understand the initial mechanism of super-enhancer activation, we used the CRISPR-Cas9 genome editing technique to establish mutant mice carrying a single deletion mutation at the ELF5 binding site; double mutations at ELF5 and STAT5 binding sites; or triple mutations at ELF5, STAT5, and NFIB binding sites in the E1 enhancer of the *Wap* super-enhancer. Our results clearly demonstrated that molecular interactions among mammary-enriched transcription factors are different in mammary epithelial cells compared with non-mammary cells and that formation of these transcription factor complexes is essential for activating the mammary-specific *Wap* super-enhancer. These findings provide a basis for understanding the mechanisms underlying mammary-specific super-enhancer establishment and offer insights that should aid in the design of strategies for cell-type-specific regeneration and treatment of diseases caused by aberrant forms of super-enhancers.

## 2. Results

### 2.1. Cellular Localization of Mammary-Enriched Transcription Factors Constituting the Wap Super-Enhancer in Mammary Epithelial Cells

**Figure 1:**
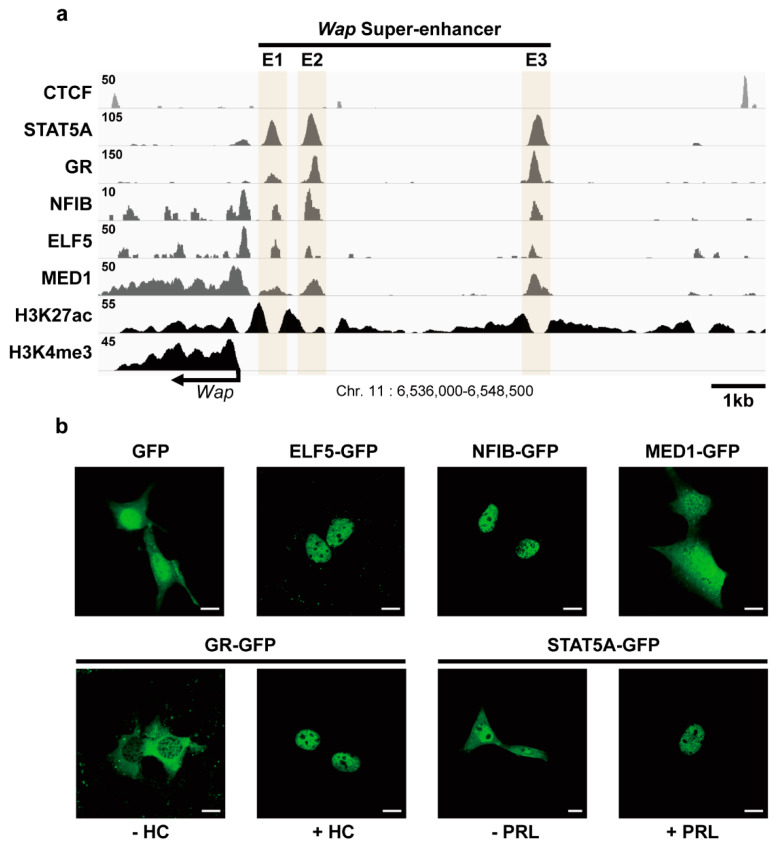
Characterization of mammary-enriched transcription factors constituting the *Wap* super-enhancer. (**a**) Genomic features of the super-enhancer in the *Wap* locus. ChIP-seq profiles of the transcriptional insulator, CTCF; the mammary-enriched transcription factors, STAT5A, GR, NFIB, and ELF5; the mediator, MED1; and the active histone marks, H3K27ac and H3K4me3. (**b**) Cellular localization of GFP-linked ELF5, NFIB, MED1, GR, and STAT5A in HC11 mammary epithelial cells. Representative fluorescence images from three independent experiments are shown (800×). After serum starvation, cells were left untreated or were treated with hydrocortisone (HC) or prolactin (PRL) to activate GR or STAT5A, respectively. Scale bars, 10 μM.

Previous ChIP-seq analyses have revealed that a super-enhancer associated with the *Wap* gene consists of three constituent enhancers: the proximal E1 enhancer (−0.7 kb from the transcription start site (TSS)), the distal E2 enhancer (−1.4 kb from the TSS), and the E3 enhancer (−5.6 kb from the TSS) (Figure 1a) [6]. Like other super-enhancers, the *Wap* super-enhancer is sequestered by the insulator CTCF, and the promoter of the *Wap* gene is highly enriched with H3K4me3 histone marks [25,26]. Each constituent enhancer is co-occupied by transcription factors essential for mammary gland development (STAT5, GR, NFIB, and ELF5), the mediator MED1, and transcription-activating H3K27ac enhancer marks. To investigate whether co-occupancy of each transcription factor within the *Wap* super-enhancer reflects independent chromatin binding of individual factors or is attributable to interactions with other transcription factors, we first determined the cellular localization of each transcription factor in mammary epithelial cells. To this end, murine full-length ELF5, NFIB, GR, STAT5A, and MED1 were tagged with green fluorescent protein (GFP) and their cellular localizations in both mammary epithelial HC11 cells and non-mammary NIH3T3 cells were visualized by fluorescence microscopy (Figure 1b, Supplementary Figure S1). ELF5 and NFIB were exclusively localized to the nucleus, whereas MED1 was localized to both the nucleus and cytoplasm. GR was mostly limited to the cytoplasm but gained entrance to the nucleus after stimulation with hydrocortisone (HC). STAT5A was localized to both the nucleus and cytoplasm but translocated to the nucleus following stimulation with prolactin (PRL). These data indicate that the transcription factors constituting the *Wap* super-enhancer can independently enter the nucleus in both mammary epithelial and non-mammary cells.

### 2.2. Protein Interactions between Mammary-Enriched Transcription Factors in Mammary Epithelial Cells Differ from Those in Non-Mammary Cells

**Figure 2:**
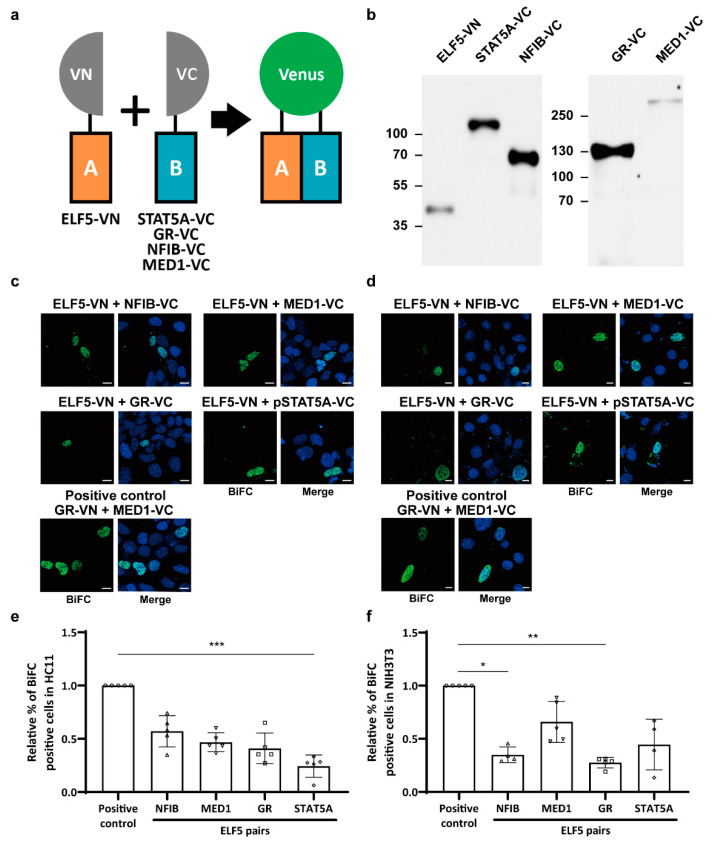
Comparison of in vivo molecular interactions between mammary-enriched transcription factors in mammary epithelial cells and non-mammary cells. (**a**) Schematic illustration of plasmid constructs and the principle of the BiFC assay used to detect protein–protein interactions. ELF5 was tagged with the N-terminal domain of the Venus protein (VN), and STAT5A, GR, NFIB, and MED1 were tagged with the C-terminal domain of the Venus protein (VC). Once VN- and VC-tagged proteins are close enough to interact, the Venus protein becomes reconstituted and emits fluorescence from cells. (**b**) Protein expression of Venus-tagged ELF5, STAT5A, NFIB, GR, and MED1, detected by Western blot analysis. (**c**,**d**) Fluorescence images obtained from a BiFC assay in (**c**) mammary epithelial HC11 cells (800×) and (**d**) mouse fibroblast NIH3T3 cells (600×). BiFC signals are shown in green, and DAPI-stained cell nuclei are shown in blue. The GR-VN and MED1-VC pair was used as a positive control. Representative images from five independent experiments are shown. Scale bars, 10 μM. (**e**,**f**) Relative percentages of BiFC-positive (**e**) HC11 cells and (**f**) NIH3T3 cells. The number of BiFC-positive cells was normalized to that of DAPI-stained cells in three different fields per slide from five independent experiments (* *p* < 0.05; ** *p <* 0.01; *** *p* < 0.001). A circle, a positive control; a triangle, ELF5-NFIB; an inverted triangle, ELF5-MED1; a square, ELF5-GR; a diamond, ELF5-STAT5A.

A previous study using mutant mice revealed that ELF5 plays an important role in activating the *Wap* super-enhancer [6]. Mutating a single STAT5 binding site or both STAT5 and NFIB binding sites in the E1 enhancer was not sufficient to disrupt *Wap* super-enhancer activity, but mutating all three STAT5, NFIB, and ELF5 binding sites completely disabled super-enhancer function. To determine whether ELF5 can interact with the four other transcription factors—STAT5A, NFIB, GR, and MED1—constituting the *Wap* super-enhancer, we investigated in vivo protein–protein interactions in mammary epithelial cells using the BiFC assay, which is designed to assess interactions between two fluorescence-tagged proteins in vivo [19,20,21]. Briefly, when the target protein linked to the N-terminal domain of a fluorescent protein and the counterpart molecule linked to the C-terminal domain of the same fluorescent protein come sufficiently close to each other to molecularly interact, the N- and C-terminal domains of distinct fluorescent proteins bind to each other to form a complex, subsequently emitting intracellular fluorescence. To apply this strategy, we linked the N-terminal domain of the Venus fluorescent tag (VN) to ELF5 and the C-terminal domain of the Venus fluorescent tag (VC) to STAT5A, GR, NFIB, or MED1 (Figure 2a). Prior to BiFC assays, we confirmed that each fusion protein was properly expressed in cells (Figure 2b). We next investigated protein–protein interactions between ELF5 and the other four transcription factors, STAT5A, GR, NFIB, and MED1, in mammary epithelial HC11 cells and non-mammary NIH3T3 cells. Since GR and MED1 are known to interact with each other [27], we used the GR-VN and MED1-VC pair as a positive control for the BiFC assay. As expected, the positive control (GR-VN and MED1-VC pair) emitted strong Venus fluorescence in both HC11 and NIH3T3 cells, whereas the negative control (JUN-VN and ELF5-VC pair) did not emit a fluorescence signal in either cell type (Supplementary Figure S2). Although ELF5 paired with each of the four other transcription factors induced fluorescence in both HC11 and NIH3T3 cells (Figure 2c,d), the patterns of molecular interactions in mammary epithelial cells and non-mammary cells were completely different (Figure 2e,f). In HC11 cells, the percentage of BiFC-positive cells for protein pairs followed the rank order ELF5–NFIB, ELF5–MED1, ELF5–GR, and ELF5–STAT5A (Figure 2e), indicating that ELF5 can interact with all four transcription factors, but with slightly different affinities. Non-mammary NIH3T3 cells showed different BiFC patterns compared with mammary epithelial cells, with ELF5 exhibiting the strongest interaction with MED1 and the weakest interaction with GR (Figure 2f). Collectively, these data suggest that the pattern of molecular interactions within a mammary-enriched transcription factor complex differs between mammary epithelial cells and non-mammary cells, a difference that may form the basis of the unique features of mammary-specific super-enhancers.

**Figure 3:**
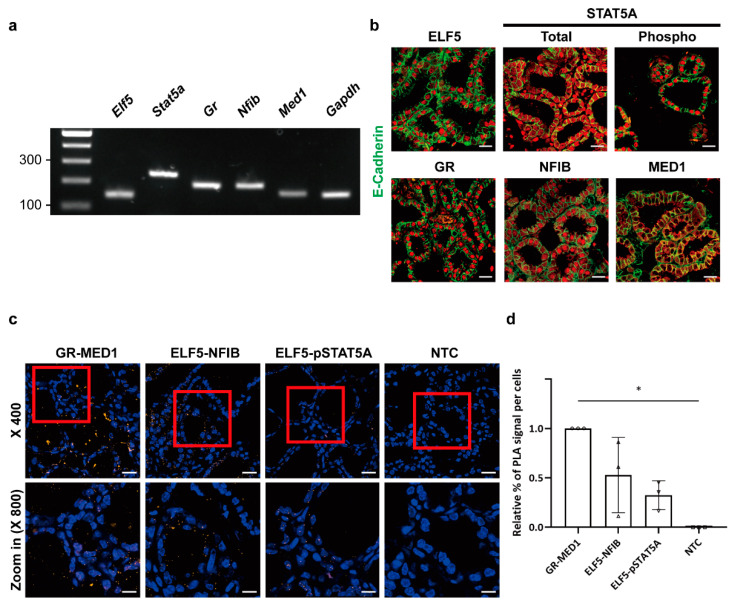
In situ protein–protein interactions of mammary-enriched transcription factors detected by PLA in lactating mouse mammary epithelium. (**a**) Endogenous expression levels of *Elf5*, *Stat5a*, *Gr*, *Nfib*, *Med1*, and *Gapdh* genes in mouse mammary tissue obtained at L1. mRNA levels of each gene were measured by RT-qPCR. (**b**) Immunofluorescence of ELF5, STAT5A, GR, NFIB, and MED1 proteins in mouse mammary tissue obtained at L1 (400×). Each transcription factor is stained in red, and the epithelial marker E-cadherin is stained in green. pSTAT5A was detected using an anti-phospho-STAT5 antibody. Representative images from three independent experiments are shown. Scale bars, 20 μM. (**c**) PLA signals in mouse mammary tissue at L1 visualized at low magnification (400×; scale bar, 20 μM) and high magnification (800×; scale bar, 10 μM). PLA signals are shown in orange, and DAPI-stained cell nuclei are shown in blue. The GR and MED1 pair was used as a positive control. Representative images from three independent replicates are shown. (**d**) Relative percentage of PLA signals per cell. The number of PLA puncta in DAPI-stained nuclei was counted from five different fields in each slide from three different donor mice (* *p* < 0.05). NTC, non-treated control. A circle, GR-MED1; a triangle, ELF5-NFIB; an inverted triangle, ELF5-pSTAT5A; a square, NTC.

To further confirm the molecular interactions of ELF5 with NFIB, MED1, GR, and STAT5A, we performed in situ PLAs in the mammary epithelium of lactating mice. PLAs, which integrate immunoassay and fluorescence microscopy techniques, are useful for investigating protein–protein interactions in unmodified cells or tissue [22,23,24]. In application, the target protein and its counterpart in tissues are immunoprecipitated with the corresponding primary antibodies and PLA probe-linked secondary antibodies. If two proteins come sufficiently close to molecularly interact, a specific DNA strand attached to the PLA probe is capable of initiating rolling circle DNA synthesis. The DNA circle is subsequently amplified several hundred fold and binds to a fluorescently labeled complementary oligonucleotide probe, achieving a high concentration of fluorescence that can be visualized by fluorescence microscopy. Before conducting PLA assays, we used quantitative reverse transcription polymerase chain reaction (RT-qPCR) to confirm the endogenous expression of *Elf5*, *Stat5a*, *Gr*, *Nfib*, and *Med1* in mouse mammary epithelium obtained on day 1 of lactation (L1) (Figure 3a). Immunofluorescence assays also revealed the expression of ELF5, STAT5A, GR, NFIB, and MED1 proteins in lactating mouse mammary epithelium (Figure 3b). Consistent with BiFC results, ELF5 showed stronger interactions with NFIB than with phosphorylated STAT5A (pSTAT5A) in mouse epithelium (Figure 3c). The GR and MED1 positive control pair exhibited strong PLA signals, whereas untreated cells showed no fluorescence signal. The relative percentage of PLA signals per cell was higher for the ELF5 and NFIB pair than for the ELF5 and pSTAT5A pair (Figure 3d). Taken together, in vivo BiFC assays and in situ PLAs demonstrated that ELF5 can form a protein complex with NFIB, GR, MED1, and pSTAT5A, but with different affinities. ELF5 interacts more closely with NFIB than with STAT5A in mammary epithelium, whereas ELF5 is more closely associated with MED1 in non-mammary cells.

### 2.3. Mammary-Enriched Transcription Factors Synergize to Activate the Wap Super-Enhancer for Mammary Gland Differentiation

**Figure 4:**
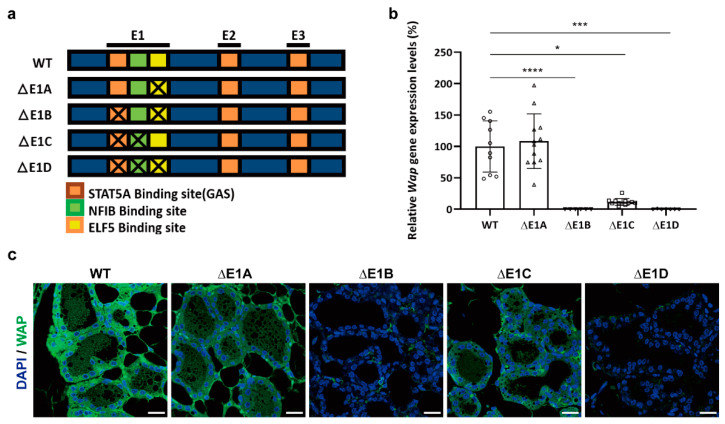
Expression of the *Wap* gene in E1-mutant mice. (**a**) Schematic depiction of CRISPR-Cas9-targeted mice. (**b**) Relative expression levels of the *Wap* gene in E1-mutant mice at L1. *Wap* mRNA levels were measured by RT-qPCR and normalized to *Gapdh* levels. Data are presented as means ± SD of different donor mice for each mouse line (* *p* < 0.05; *** *p* < 0.001; **** *p* < 0.0001). WT, n = 10; ΔE1A, n = 12; ΔE1B, n = 6; ΔE1C, n = 12; ΔE1D, n = 7. A circle, WT; a triangle, ΔE1A; an inverted triangle, ΔE1B; a square; ΔE1C; a diamond, ΔE1D (**c**) Immunofluorescence images of WAP expression in L1 mammary tissues of mutant mice. WAP is shown in green, and DAPI-stained cell nuclei are shown in blue. Representative images from three different donor mice for each mouse line are shown. Magnification, 400×; scale bar, 20 μM.

A previous study using CRISPR-Cas9-targeted mice revealed that mutations of STAT5, NFIB, and ELF5 binding sites in the E1 enhancer decommission the *Wap* super-enhancer [6]. However, whether all three transcription factors are required for the establishment of a super-enhancer complex or whether ELF5 plays a pioneering role in initiating the activation of a super-enhancer has not been clearly elucidated. To address this issue, we established CRISPR-Cas9-targeted mice carrying the following deletion mutations in the E1 enhancer of the *Wap* super-enhancer: deletion of a single ELF5 binding site (ΔE1A); combined deletion of ELF5 and STAT5 binding sites (ΔE1B); combined deletion of STAT5 and NFIB binding sites (ΔE1C); and combined deletion of ELF5, STAT5, and NFIB binding sites (ΔE1D) (Figure 4a and Supplementary Figure S3). We first assessed *Wap* gene expression levels in these E1 mutant mice (Figure 4b). Consistent with previous results [6], deletion of all three ELF5, NFIB, and STAT5 binding sites (ΔE1D) completely silenced *Wap* gene expression in mice. Although expression levels of the *Wap* gene in mice with a single ELF5 binding site deletion (ΔE1A) were comparable to those in wild-type mice, deletion of ELF5 and STAT5 binding sites (ΔE1B) reduced *Wap* gene expression levels by 99.9% compared with wild-type controls, and deletion of STAT5 and NFIB binding sites (ΔE1C) decreased *Wap* gene expression levels by 88% compared with wild-type mice. Immunofluorescence analysis also confirmed that WAP proteins were rarely expressed in mammary alveolar cells of ΔE1B and ΔE1D mice at L1 (Figure 4c). WAP proteins were abundantly expressed in lactating mammary alveoli in ΔE1A mice and modestly expressed in ΔE1C mice. Expression levels of *Csn2*, a gene encoding another milk protein, were not affected by mutations at E1 enhancers in mice (Supplementary Figure S4). Collectively, our findings indicate that mutation of a single transcription factor binding site at E1 is not sufficient to disable the *Wap* super-enhancer function. Instead, the bindings of all three transcription factors—ELF5, STAT5, and NFIB—at the E1 enhancer synergize to constitute the full function of the *Wap* super-enhancer.

### 2.4. Protein–Protein Interactions within a Mammary-Enriched Transcription Factor Complex Are Essential for Activation of Mammary-Specific Wap Super-Enhancers

**Figure 5:**
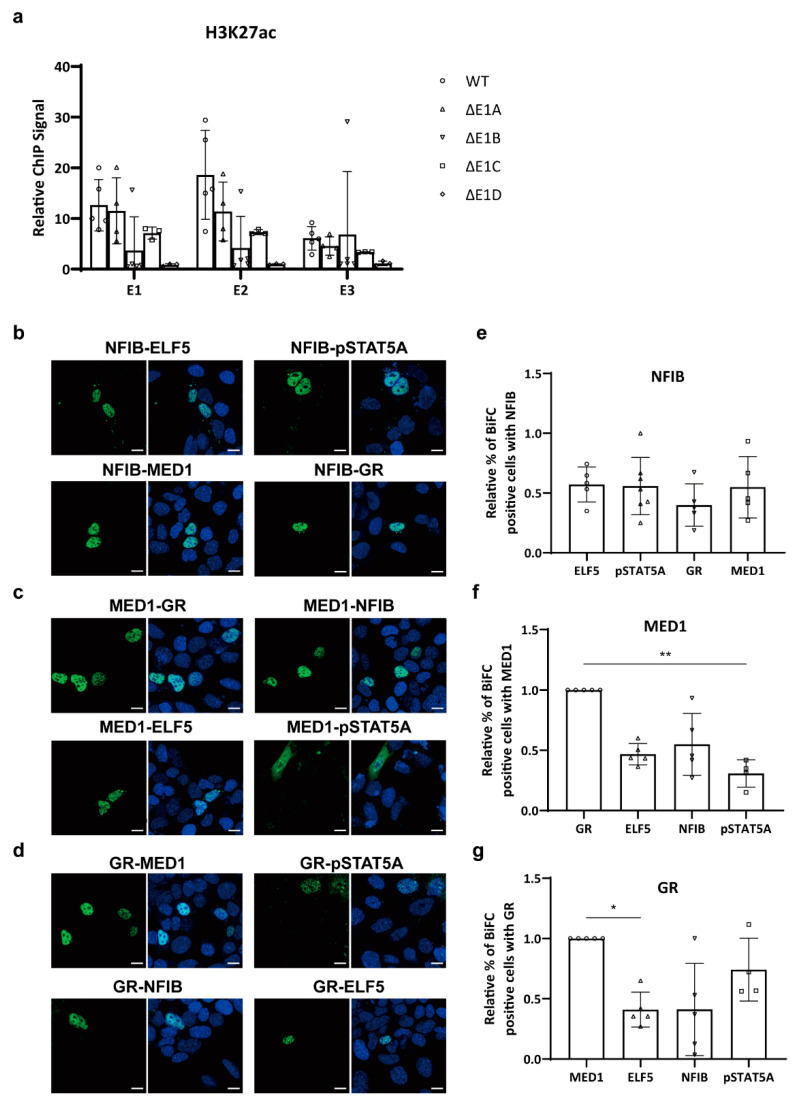
Chromatin features of E1, E2, and E3 constituent enhancers within the mammary-specific *Wap* super-enhancer. (**a**) ChIP analyses of the active enhancer mark, H3K27ac, in E1-mutant mice. Relative ChIP signals are shown. WT, n = 5; ΔE1A, n = 4; ΔE1B, n = 5; ΔE1C, n = 3; ΔE1D, n = 3. ChIPed DNA levels were normalized to corresponding input DNA levels. (**b**–**d**) Molecular interactions between mammary-enriched transcription factors in HC11 cells, detected by BiFC analyses. BiFC signals are shown in green, and cell nuclei are shown in blue. Magnification, 800×; scale bar, 10 μM. Representative images from five independent experiments are shown. (**e**–**g**) Relative percentages of BiFC-positive cells. The number of BiFC-positive cells was normalized to that of DAPI-stained cells in three different fields per slide from five independent experiments (* *p* < 0.05; ** *p* < 0.01).

We further investigated whether the limited *Wap* gene expression in ΔE1B and ΔE1D mice was attributable to the inactivation of all three constituent *Wap* super-enhancers, E1, E2, and E3. To this end, we performed ChIP assays to detect modifications in H3K27ac, a histone mark typically enriched at active enhancer regions. Consistent with observed *Wap* gene expression levels, H3K27ac levels at the E1 enhancer of ΔE1A mice were comparable to those of wild-type controls and were reduced by ~40% in ΔE1C mice (Figure 5a). On the other hand, H3K27ac levels at the E1 enhancer were reduced by ~70% in ΔE1B mice and by ~90% in ΔE1D mice. H3K27ac signals on E2 and E3 enhancers revealed that E2 and E3 were modestly activated in ΔE1A mice, whereas a dysfunctional E1 enhancer suppressed the activation of distal E2 and E3 enhancers in ΔE1B, ΔE1C, and ΔE1D mice.

In addition to investigating the molecular interactions between ELF5 and the transcription factors NFIB, GR, MED1, and pSTAT5A, we also evaluated protein interactions between each transcription factor pair to further estimate the molecular proximity within the protein complex recruited to the *Wap* super-enhancer (Figure 5b–d). NFIB interacted with ELF5, pSTAT5A, and MED1 to a similar degree and showed slightly weaker interactions with GR (Figure 5e); MED1 showed the strongest binding to GR and the weakest interaction with pSTAT5A (Figure 5f); and GR bound most strongly to MED1 and least strongly to NFIB and ELF5 (Figure 5g). These data indicate that all five transcription factors (ELF5, NFIB, GR, MED1, and pSTAT5A) can interact with each other, but their differential binding intensities suggest differences in molecular proximity within the protein complex. BiFC results shown in Figure 3 and Figure 5 suggest that MED1 mediates protein interactions between the ELF5–NFIB complex and the GR–pSTAT5A complex.

## 3. Discussion

Super-enhancers were first discovered in embryonic stem cells about a decade ago [7,8]. Since then, super-enhancers have been identified in a variety of cell types, including mammary epithelial cells, immune cells, chondrocytes, and hair follicle cells [6,13,28,29]. Accumulating evidence also shows that abnormal forms of super-enhancers are causally linked to various diseases [7,11,12,13]. However, the detailed molecular interactions within a transcription factor complex recruited on super-enhancers have not been elucidated. Although several super-enhancer studies have identified the pioneering factor that establishes super-enhancers in specific cell types [29], the initial activation mechanism of mammary-specific super-enhancers has not been investigated. Here, we used both in vivo BiFC assays and in situ PLAs to unravel the protein interactions between mammary-enriched transcription factors that constitute mammary-specific super-enhancers. Our results clearly revealed that the pattern of molecular interactions of these transcription factors differs between mammary epithelial cells and non-mammary cells, underscoring the unique characteristics of cell-type-specific super-enhancers. We further created several CRISPR-Cas9-targeted mice for use in identifying key transcription factors essential for the initial activation of mammary-specific *Wap* super-enhancers. By disrupting binding sites for key transcription factors at the E1 pioneering enhancer, we demonstrated that the mammary-specific *Wap* super-enhancer is activated by synergy among key transcription factors co-localized at the pioneering enhancer rather than by a single molecule (Supplementary Figure S5). The present study also revealed that disruption of all three binding motifs, ELF5, NFIB, and STAT5, in the E1 enhancer markedly reduced the acetylation of H3K27 in the distal E2 and E3 enhancers, whereas deletion of the ELF5 and STAT5 binding motifs from the E1 enhancer preferentially inhibited the activity of the E2 enhancer. Deletion of STAT5 and NFIB from the E1 enhancer modestly reduced activation of the E2 and E3 enhancers, whereas deletion of a single STAT5 motif had minimal effect on these distal enhancers. Additional studies are required to determine whether these differential effects are caused by chromatin looping between the proximal E1 enhancer and the distal E2 and E3 enhancers.

Several studies have shown that super-enhancers are typically bound by lineage-specific transcription factors, and a single pioneering factor is essential for their activation [8,29]. Oct-4 and Sox9 play a pioneering role in the activation of super-enhancers in embryonic stem cells and hair follicle cells, respectively. Unlike these super-enhancers, the mammary-specific *Wap* super-enhancer appears to be activated by cooperation among the key transcription factors ELF5, STAT5, NFIB, GR, and MED1, which are essential for mammary gland development. Therefore, the cell-type-specific molecular interactions between major transcription factors might be critical for the unique properties of mammary-specific super-enhancers. Our results also suggest that ELF5 and STAT5 play a critical role in the pioneering E1 enhancer of the mammary-specific *Wap* super-enhancer. Although we could estimate the molecular proximity between transcription factors that constitute mammary-specific super-enhancers using in vivo BiFC assays and in situ PLAs, these techniques may not be sufficient to investigate the molecular interplay within a native protein complex bound to a super-enhancer. A future study using ChIP-mass spectrophotometry may be necessary to accurately determine the molecular interactions in intact super-enhancer complexes [30]. Recent studies have also reported evidence for the formation of phase-separated condensates—microscopically detectable transcriptional regulatory complexes [31,32]—in super-enhancer regions. An intriguing question is whether such phase-separated condensates can also be found in mammary epithelial cells. Because mammary gland epithelium undergoes repeated alveolar formation and degeneration, it will be an ideal model for understanding super-enhancer assembly and disassembly processes, which would explain the reversible feature of super-enhancers. Moreover, these findings and experimental approaches may be useful for studying the molecular mechanisms of super-enhancer activity in different breast cancer subtypes. ChIP-seq analyses found that super-enhancers in estrogen receptor (ER)-positive breast cancer cells co-localize with ELF5, FOXA1, and ER [33]. In contrast, super-enhancers found in triple-negative breast cancer (ER^−^, PR^−^, HER2^−^) co-localized with the transcription factors FOXC1, MET, MYC, and ANLN [34,35]. It will be interesting to determine whether mammary-enriched ELF5, STAT5, NFIB, and GR are also recruited in these super-enhancers and whether differential molecular recruitment to super-enhancers determines the different breast cancer subtypes. Our findings offer basic insight into the regulatory mechanisms underlying mammary-specific developmental processes and further suggest how this knowledge might be applied to cell-type-specific regeneration and treatment of abnormal super-enhancer-driven diseases.

## 4. Materials and Methods

### 4.1. ChIP-Seq Data

All ChIP-seq data were obtained from datasets deposited in the Gene Expression Omnibus (GEO) under accession number GSE74826 [6] and were visualized with an Integrated Genomics Viewer (IGV version 2.8.9., Broad Institute and the Reagents of the University of California, CA, USA).

### 4.2. Plasmids Construction

GFP-tagged constructs were generated by individually cloning murine full-length ELF5, STAT5A, NFIB, GR, and MED1 cDNAs into AcGFP-C1 (#54607; Addgene, Watertown, MA, USA). The Venus N-terminal domain (VN)-tagged construct was created by PCR-amplifying ELF5 from ELF5-GFP and cloning it into pBiFC-VN155 (I152L) (#27097; Addgene). Venus C-terminal domain (VC)-tagged constructs were generated by PCR-amplifying STAT5A, NFIB, GR, and MED1 from the corresponding GFP-tagged constructs and cloning them individually into pBiFC-VC155 (#22011; Addgene).

### 4.3. Cell Culture

The mouse mammary epithelial HC11 cell line was purchased from the American Type Culture Collection (ATCC, Manassas, VA, USA), and the mouse embryonic fibroblast NIH3T3 cell line was kindly provided by Dr. Young Bong Kim (Konkuk University, Seoul, Korea). HC11 cells were maintained in Roswell Park Memorial Institute 1640 medium (RPMI 1640; Capricorn Scientific, Ebsdorfergrund, Germany) supplemented with 10% fetal bovine serum (FBS; Merck, Kenilworth, NJ, USA) and 1% penicillin/streptomycin (Capricorn Scientific). NIH3T3 cells were cultured in Dulbecco’s Modified Eagle’s Medium (DMEM; Capricorn Scientific) supplemented with 10% FBS and 1% penicillin/streptomycin. Both cell lines were grown in a humidified incubator at 37 °C under 5% CO_2_ conditions.

### 4.4. Western Blot Analysis

Cells were lysed with cold RIPA buffer (150 mM NaCl, 50 mM Tris pH 7.5, 0.1% SDS, 1% Triton X-100, 0.5% deoxycholate, protease inhibitor cocktail (Roche, Basel, Switzerland), 1 mM orthovanadate, 10 mM NaF, 100 mM PMSF) with gentle sonication. After protein concentration in cleared lysates (supernatants) was measured, proteins were denatured in sodium dodecyl sulfate (SDS) sample buffer and separated by SDS-PAGE on 10% gels. For Western blot analysis, proteins were transferred to a nitrocellulose membrane and then incubated first with an anti-GFP primary antibody (#ab6556; Abcam, Cambridge, UK) and then with a horseradish peroxidase (HRP)-conjugated anti-mouse IgG (#A10551; Invitrogen, Waltham, MA, USA) or HRP-conjugated anti-rabbit IgG (#G21234; Invitrogen) secondary antibody as appropriate. Immunoreactive proteins were detected using an enhanced chemiluminescence system.

### 4.5. Bimolecular Fluorescence Complementation (BiFC) Assay

Cells were plated on glass coverslips in 12-well plates containing growth medium 1 day before transfection. When cells reached ~70% confluence, they were transfected with BiFC constructs using the Lipofectamine 3000 reagent (Invitrogen) according to the manufacturer’s instructions. After 24 h of serum starvation, cells were treated with prolactin (PRL) or hydrocortisone (HC) for 1 h to activate STAT5 or GR, respectively. Cells were washed with phosphate-buffered saline (PBS) and fixed with ice-cold 4% paraformaldehyde in PBS for 15 min. Fixed cells were mounted on glass slides with ProLong Gold Antifade Mountant containing DAPI (Invitrogen). GFP- and Venus-tagged proteins were visualized with a Zeiss LSM 800 confocal microscope using a 40× oil-immersion objective (Zeiss, Oberkochen, Germany). GFP and Venus were excited at 509 nm using an argon laser, and emission was collected using a 400–650 nm filter. DAPI was excited at 465 nm using an argon laser, and emission was collected using a 400–495 nm filter. Images were captured using the ZEN image browser (ZEN blue version 2.6; Zeiss).

### 4.6. Mice

Eight-week-old female C57BL/6 mice were purchased from Orient Bio (Seongnam, Korea) and used as wild-type controls. Founder CRISPR-Cas9-targeted mice were generated by Toolgen (Seoul, Korea). Single-guide RNAs (sgRNAs) were designed to specifically target the ELF5 motif or the region spanning the GAS to ELF5 motif at E1 of the *Wap* super-enhancer (Supplementary Table S1). All mice were maintained under specific-pathogen-free conditions with a 12 h light/dark cycle at 20–25 °C (room temperature) and 30–70% relative humidity. All animal procedures were approved by the Institutional Animal Care and Use Committee (IACUC) of Konkuk University (Seoul, Korea).

### 4.7. Generation of Homozygous Mice and Genotyping

Founder (F0) CRISPR-Cas9-targeted mice were bred with C57BL/6 wild-type mice to segregate the mosaicism, resulting in the generation of F1 heterozygous mice. F2 homozygous mice were generated by interbreeding F1 mice. All mice were genotyped by PCR amplification of genomic DNA isolated from mouse tail snips, followed by Sanger sequencing. Details of the PCR primers and sequencing primers are presented in Supplementary Table S2. The four lines carrying deletions in transcription factor binding sites at E1 (0.7 kb upstream of the *Wap* TSS) were named ΔE1A (9-bp deletion), ΔE1B (49-bp deletion), ΔE1C (26-bp deletion), and ΔE1D (97-bp deletion). The specific sequences deleted in each mouse are provided in Supplementary Figure S3.

### 4.8. Histological Analysis

Mammary tissues were harvested on day 1 of lactation (L1), fixed in 10% formalin, dehydrated using an ethanol series and xylene, and embedded in paraffin according to standard protocols. Paraffin blocks were sectioned at 4 μm thickness. For immunohistochemistry, antigen unmasking was performed in a TintoRetriever Heat Retrieval System (Bio SB Inc., Goleta, CA, USA) using 10 mM sodium citrate buffer (pH 6.0) for 10 min. Sections were blocked by incubating with 3% normal goat serum at room temperature for 1 h. Tissues were incubated overnight at 4 °C with the following primary antibodies (diluted 1:100): anti-STAT5A (#LS-C386212-100; LSBio, Seattle, CA, USA); anti-ELF5 (#70R-49702; Fitzgerald, Kampenhout, Belgium); anti-NFIB (#39091; Active Motif, Carlsbad, CA, USA); anti-GR (#PA1-511A; Invitrogen); anti-MED1 (#A300-793A; Bethyl Laboratories, Waltham, MA, USA); and anti-E-cadherin (#610181; BD Biosciences, Franklin Lakes, NJ, USA). After washing, tissues were incubated with Alexa Fluor 488-conjugated anti-mouse IgG (#R37120; Invitrogen) or Alexa Fluor 594-conjugated anti-rabbit IgG (#R37117; Invitrogen) for 1 h at room temperature. Slides were then mounted with ProLong Gold Antifade Mountant with DAPI (Invitrogen), imaged under a Zeiss LSM 800 confocal microscope, and analyzed using the ZEN image browser.

### 4.9. In Situ Proximity Ligation Assay (PLA)

PLA was performed with the Duolink detection kit (#DUO92103; Merck, Darmstadt, Germany). The procedure from tissue harvesting to antigen retrieval was the same as that for conventional immunochemistry, described above. Following antigen retrieval, slides were blocked by incubating at 37 °C for 30 min and then were incubated with primary antibody overnight at 4 °C. The primary antibodies used in PLA were the same as those used for histology. After washing, tissues were incubated with Plus and Minus Duolink probes, ligated, and subjected to rolling circle amplification according to the manufacturer’s protocol. Samples were mounted using ProLong Gold Antifade Mountant with DAPI (Invitrogen). PLA signals were visualized with a Zeiss LSM 800 confocal microscope and analyzed using the ZEN image browser. PLA signals were detected using the Cy3 filter (excitation, 561 nm; emission, 535–700 nm), and nuclei were visualized with the DAPI filter (excitation, 465; emission, 400–600 nm). PLA signals were counted in cell nuclei in five different fields per slide from three independent replicates.

### 4.10. Quantitative (RT-qPCR) and Conventional RT-PCR

RNA was extracted using the PureLink RNA Mini Kit (Invitrogen) according to the manufacturer’s instructions. cDNA was synthesized from total RNA by reverse transcription using SuperScript III Reverse Transcriptase (Invitrogen). qPCR was then performed on a LightCycler 96 Instrument (#05815916001; Roche, Basel, Switzerland) using the SsoAdvanced Universal SYBR Green Supermix (BioRad, Hercules, CA, USA) and gene-specific primers. Sequences of primers used for RT-qPCR are listed in Supplementary Table S3. *Wap* and *Csn2* mRNA levels were normalized to *Gapdh* levels. Conventional RT-PCR was carried out using specific primers for *Elf5*, *Stat5a*, *Gr*, *Nfib*, *Med1*, or *Gapdh* (internal control). Sequences of primers used for conventional RT-PCR are provided in Supplementary Table S4.

### 4.11. Chromatin Immunoprecipitation (ChIP) Analysis

Frozen, stored mammary tissues harvested at L1 were ground into a powder using a mortar and pestle. Chromatin was fixed by incubation with 1% formaldehyde for 10 min at room temperature; fixation was stopped by adding glycine (final concentration, 125 mM). Nuclei were extracted with Farnham Lysis Buffer (5 mM PIPES pH 8.0, 85 mM KCl, 0.5% NP-40) supplemented with PMSF and proteinase inhibitor cocktail. Chromatin was fragmented to 200–400 bp using a VCX-130 Vibra-Cell sonicator (60 cycles; 30-s pulse/30-s rest; Sonics, Newtown, CT, USA) followed by lysis with RIPA buffer. Chromatin (1 mg) was immunoprecipitated with Dynabeads Protein A (Invitrogen) coated with anti-H3K27ac antibody (#ab4729; Abcam, Cambridge, UK). Beads were serially washed with low-salt buffer (150 mM NaCl, 20 mM Tris–HCl pH 8.0, 0.1% SDS, 1% Triton X-100, 2 mM EDTA), high-salt buffer (500 mM NaCl, 20 mM Tris–HCl pH 8.0, 0.1% SDS, 1% Triton X-100, 2 mM EDTA), LiCl wash buffer (0.25 M LiCl, 10 mM Tris–HCl pH 8.0, 1% NP-40, 1% sodium deoxycholate, 1 mM EDTA), and PBS. After reverse-crosslinking at 65 °C overnight with elution buffer in the presence of 1% SDS and 1 mg/mL of proteinase K (ThermoFisher, Waltham, MA, USA), ChIP DNA was purified using a QIAquick PCR Purification Kit (Qiagen, Hilden, Germany). qPCR was then performed on a LightCycler 96 Instrument (Roche) using the SsoAdvanced Universal SYBR Green Supermix (BioRad). The sequences of primers used for qPCR are listed in Supplementary Table S5.

### 4.12. Statistical Analyses

Data were evaluated by one-way non-parametric ANOVA (Kruskal–Wallis test) using GraphPad Prism 9.4.0. (GraphPad Software, San Diego, CA, USA) and are presented as means ± standard deviation (SD). Prior to any statistical analysis, we conducted a Shapiro–Wilk normality test to confirm the normal distribution of the data.

## Data Availability

Any data or materials that support the findings of this study can be made available by the corresponding author upon request.

## Supplementary Materials

The following supporting information can be downloaded at https://www.mdpi.com/article/10.3390/ijms231911680/s1.
