# Supplementary material for: Mammary-Enriched Transcription Factors Synergize to Activate the Wap Super-Enhancer for Mammary Gland Development
Source: Int J Mol Sci. 2022 Oct 2;23(19):11680. doi: 10.3390/ijms231911680 (PMC9569684; doi:10.3390/ijms231911680)
Supplement: Supplementary file 1 [file ijms-23-11680-s001.zip › ijms-1896841-supplementary.pdf]

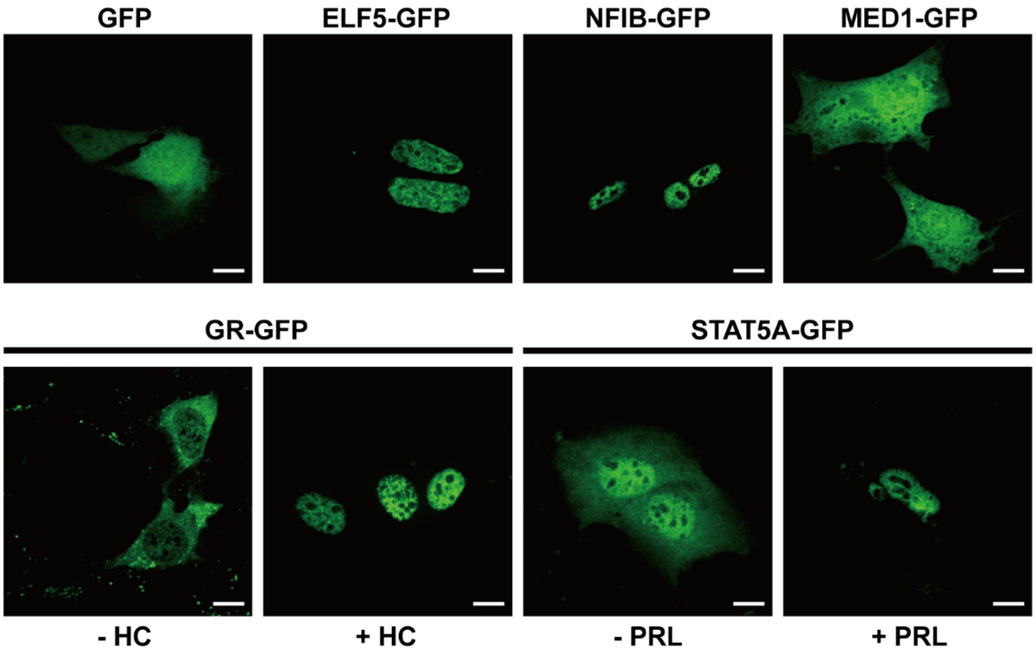

Supplementary Figure S1. Cellular localization of mammary-enriched transcription factors constituting the mammary-specific super-enhancer in NIH3T3 mouse embryonic fibroblasts. Fluorescence images of GFP-linked ELF5, NFIB, MED1, GR, and STAT5A visualized by fluorescence microscopy. After serum starvation, cells were left untreated or were treated with hydrocortisone (HC) or prolactin (PRL) to activate GR or STAT5A, respectively. Representative images from three independent replicates are shown (400x). Scale bars: 10 μm.

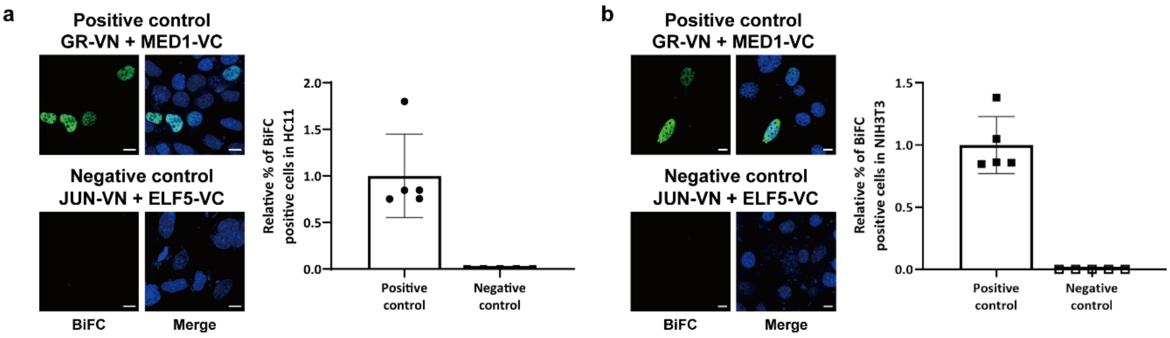

Supplementary Figure S2. BiFC analysis of control groups in HC11 and NIH3T3 cells. (a) HC11 cells were transfected with GR-VN and MED-VC as a positive control and with JUN-VN and ELF5-VC as a negative control. Representative fluorescence microscopy images are shown (800x). Scale bars: 10 μm. A circle, positive control; a square, a negative control (b) Representative fluorescence microscopy images of positive and negative controls for BiFC analyses in NIH3T3 cells (600x). Scale bars: 10 μm. A closed square, a positive control; an open square, a negative control

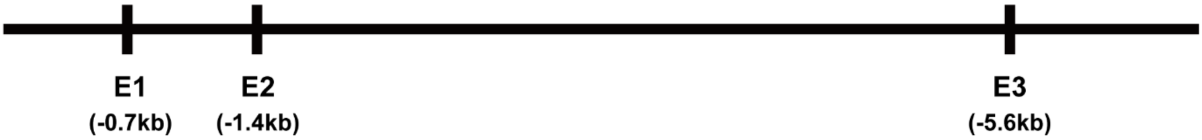

Mice	Deletion sequence at E1
**WT**	23bp<br>AAGGCCACTTCCCAGAAGGGCCCATACTGTGCCAAGG....CCTGCTGGGCACTGTGCCCCATTGTTCTCGGGACATTTCCTTGATTTC
**ΔE1A** (-9bp)	AAGGCCACTTCCCAGAAGGGCCCATACTGTGCCAAGG....CCTGCTGGGCACTGTGCCCCATTGTTCTCGGGACA---------ATTTC
**ΔE1B** (-49bp)	AAGGCCA-----CAGAAGGGCCCATACTGTGCCAAGG....CC--TTC
**ΔE1C** (-26bp)	AAG--------------------------GCCAAGG....CCTGCTGGGCACTGTGCCCCATTGTTCTCGGGACATTTCCTTGATTTC
**ΔE1D** (-97bp)	AAGGCCAC-------------------------------....---TTC

Supplementary Figure S3. Characterization of mutant mice harboring deletion mutations introduced by CRISPR-Cas9. Sequences deleted at E1 of the *Wap* super-enhancer are shown for each mouse line. Red, GAS motif; green, NFIB motif; blue, ELF5 motif.

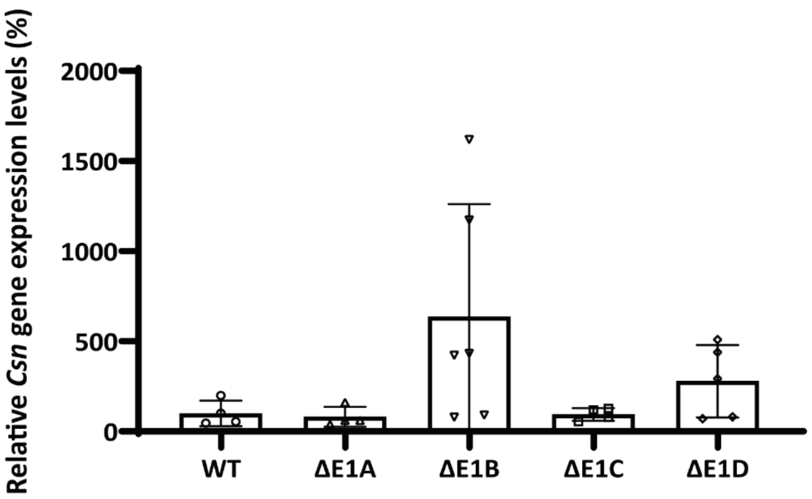

Supplementary Figure S4. Relative expression levels of the *Csn2* gene. In mutant mice on day 1 of lactation (L1). *Csn2* mRNA levels were measured by RT-qPCR and normalized to *Gapdh* levels. Results are shown as means ± SD of different donor mice for each mouse line. WT, n = 4; ∆E1A, n = 4; ∆E1B, n = 6; ∆E1C, n = 4; ∆E1D, n = 5. A circle, WT; a triangle, ∆E1A; an inverted triangle, ∆E1B; a square, ∆E1C; a diamond, ∆E1D

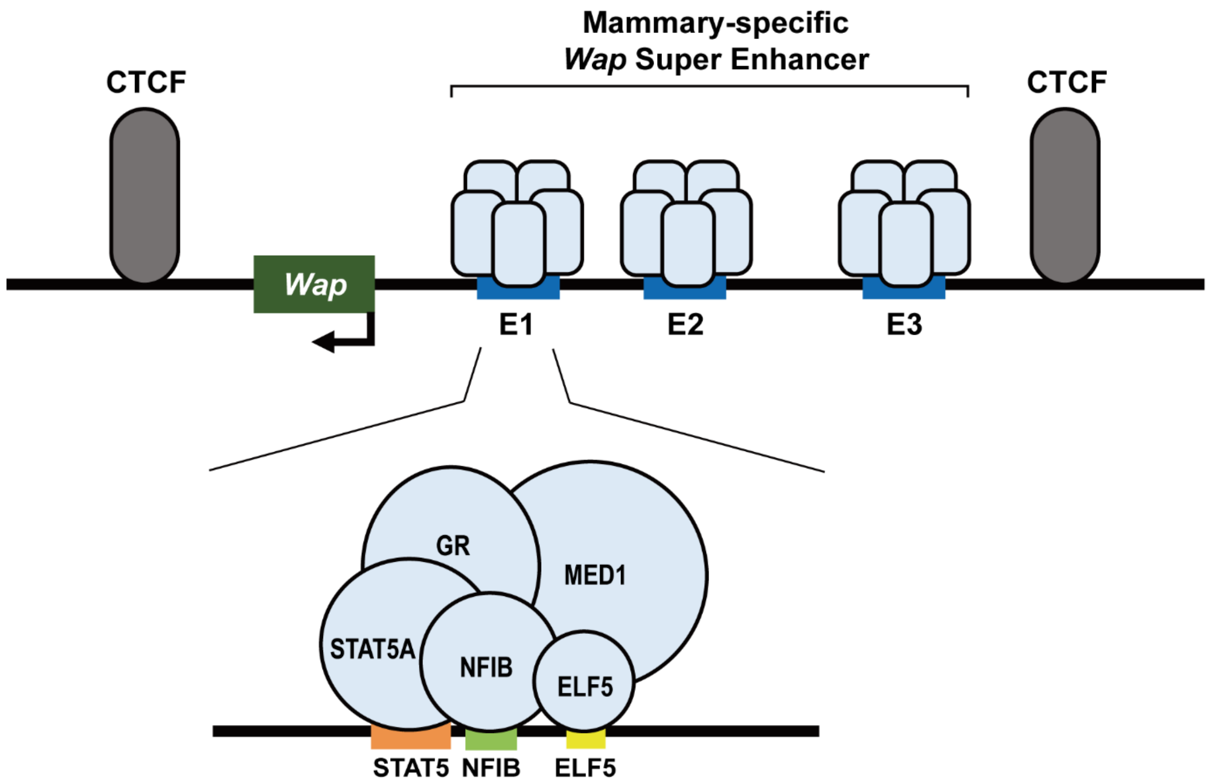

Supplementary Figure S5. Schematic illustration of the mammary-specific *Wap* super-enhancer. *Wap* super-enhancer consists of the individual enhancers E1, E2, and E3, enriched with STAT5, NFIB, ELF5, GR, and MED1. In the E1 enhancer, ELF5 is close to NFIB and STAT5 is close to GR. MED1 mediates these four transcription factors. The binding of ELF5 and STAT5 to the E1 enhancer is critical for the initiation of *Wap* super-enhancer activity, whereas an intact transcription factor complex (ELF5, STAT5, NFIB, GR, and MED1) is essential for the full function of the super-enhancer.

Supplementary Table S1. Sequences of gRNAs targeting E1 of the *Wap* super-enhancer in mice

Target motif	gRNA sequences with PAM
Elf5	5’-cttccttgcaagagaaatca AGG-3’
	5’-cactgtgccccattgttctc GGG-3’
Elf5/Nfib/Stat5	5’-cttccttgcaagagaaatca AGG-3’
	5’-gtatgggcccttctgggaag TGG-3’

Supplementary Table S2. Genotyping primers for CRISPR-Cas9–targeted mice

Purpose	Primer sequences for characterizing the E1 region	
PCR analysis	Forward	5’-ACA GGA GGT TTT GAG CAA GG-3’
	Reverse	5’-GAA ATA TCG AGG TTA CAG CC-3’
Sanger sequencing analysis	Forward	5’-TTC TTC TAA GAG TGT GGA GG-3’
	Reverse	5’-GCC ACC AGT GAA GAC AAA GG-3’

Supplementary Table S3. Gene-specific primers for RT-qPCR analyses

Target gene	Primer sequences	
Wap	Forward	5'-ATG CGT TGC CTC ATC AGC C-3'
	Reverse	5'-GAC AGG CAG GGA TGC C-3'
Csn2	Forward	5'-ACT CCA GCA TCC AGT CAC AGC-3'
	Reverse	5'-AGG TGA GTC TGA GGA AAA GCC-3'
Gapdh	Forward	5'-TGT GTC CGT CGT GGA TCT GA-3'
	Reverse	5'-TTG CTG TTG AAG TCG CAG GAG-3'

Supplementary Table S4. Gene-specific primers for semi-quantitative RT-PCR analyses

Target gene	Primer sequences	
Elf5	Forward	5'-TCT CCA GAA CAT TCG CTC GC-3'
	Reverse	5'-TTT GGA GGC TTG TTC GGC TG-3'
Stat5a	Forward	5'-AGT TTG ACT CTC CGG ACC GA-3'
	Reverse	5'-CTG TGG ATG CAT TGA CGA ACC-3'
Gr	Forward	5'-ACT GCT TCT CTC CTC AGT TCC-3'
	Reverse	5'-AGC TAA GGA GAT TTT CAA CCA CA-3'
Nfib	Forward	5'-GAA TTA CTT GGC AAA GTC CTG GT-3'
	Reverse	5'-TGG GTG TTT TTC CCC TAA TTT TCT T-3'
Med1	Forward	5'-CCC GCT GTC AGG ATG AAG G-3'
	Reverse	5'-ACG ACC CTC TTC TCC ATT ACT T-3'
Gapdh	Forward	5'-TGT GTC CGT CGT GGA TCT GA-3'
	Reverse	5'-TTG CTG TTG AAG TCG CAG GAG-3'

Supplementary Table S5. ChIP-qPCR primers for constituent *Wap* super-enhancers

Purpose	Primer sequences for characterizing the E1 region	
E1	Forward	5'-TCG GGC ATA CAT TGA AAA GG-3'
	Reverse	5'-CAC AGT CAC TCT GGG TCA TCC-3'
E2	Forward	5'-AGT CAT CCC CAC ATT TAG G-3'
	Reverse	5'-ATG CAG AGA GAA CAG AGC-3'
E3	Forward	5'-ACT GGT CAG TTG AGG ACA TG-3'
	Reverse	5'-GGA AGC ACA CAG GCT CAA G-3'
Negative control	Forward	5'-TGG CCA ATG TCT TTG TTA GG-3'
	Reverse	5'-TCA ATA CAA TGC CCA TGT CC-3'
